# Hippocampal Sclerosis Affects fMR-Adaptation of Lyrics and Melodies in Songs

**DOI:** 10.3389/fnhum.2014.00111

**Published:** 2014-02-27

**Authors:** Irene Alonso, Daniela Sammler, Romain Valabrègue, Vera Dinkelacker, Sophie Dupont, Pascal Belin, Séverine Samson

**Affiliations:** ^1^Laboratoire de Neurosciences Fonctionnelles et Pathologies (EA 4559), Université Lille-Nord de France, Lille, France; ^2^Epilepsy Unit, Hôpital de la Pitié-Salpêtrière, Paris, France; ^3^Centre de NeuroImagerie de Recherche, Groupe Hospitalier Pitié-Salpêtrière, Paris, France; ^4^Centre de Recherche de l’Institut du Cerveau et de la Moëlle Épinière, UPMC – UMR 7225 CNRS – UMRS 975 INSERM, Paris, France; ^5^Max Planck Institute for Human Cognitive and Brain Sciences, Leipzig, Germany; ^6^Centre for Cognitive Neuroimaging, Department of Psychology, University of Glasgow, Glasgow, UK; ^7^Laboratories for Brain, Music and Sound, Université de Montréal and McGill University, Montreal, QC, Canada; ^8^Institut des Neurosciences de la Timone, UMR7289, CNRS-Université Aix Marseille, Marseille, France

**Keywords:** neural adaptation, song, lyrics, hippocampal sclerosis, memory trace, conjunctive representation

## Abstract

Songs constitute a natural combination of lyrics and melodies, but it is unclear whether and how these two song components are integrated during the emergence of a memory trace. Network theories of memory suggest a prominent role of the hippocampus, together with unimodal sensory areas, in the build-up of conjunctive representations. The present study tested the modulatory influence of the hippocampus on neural adaptation to songs in lateral temporal areas. Patients with unilateral hippocampal sclerosis and healthy matched controls were presented with blocks of short songs in which lyrics and/or melodies were varied or repeated in a crossed factorial design. Neural adaptation effects were taken as correlates of incidental emergent memory traces. We hypothesized that hippocampal lesions, particularly in the left hemisphere, would weaken adaptation effects, especially the integration of lyrics and melodies. Results revealed that lateral temporal lobe regions showed weaker adaptation to repeated lyrics as well as a reduced interaction of the adaptation effects for lyrics and melodies in patients with left hippocampal sclerosis. This suggests a deficient build-up of a sensory memory trace for lyrics and a reduced integration of lyrics with melodies, compared to healthy controls. Patients with right hippocampal sclerosis showed a similar profile of results although the effects did not reach significance in this population. We highlight the finding that the integrated representation of lyrics and melodies typically shown in healthy participants is likely tied to the integrity of the left medial temporal lobe. This novel finding provides the first neuroimaging evidence for the role of the hippocampus during repetitive exposure to lyrics and melodies and their integration into a song.

## Introduction

As humans, we learn and enjoy songs from a very early age on. Over the course of our lives, we hear and remember thousands of songs and, most of the time, we learn them implicitly without much effort especially after repeated presentations (as with hit songs on the radio). Songs naturally combine music and language into a unique acoustic signal. However, it remains unclear whether memory traces of lyrics and melodies are built separately or in integration. Indeed, evidence from healthy participants and brain-damaged patients diverge on this question. On the one hand, several behavioral studies in healthy participants support the tight association of lyrics and melodies during the creation of a song memory trace as shown by cueing effects of one element on the other during song recognition (Serafine et al., 1984, 1986; Crowder et al., 1990; Baur et al., 2000; Peretz et al., 2004; Peynircioglu et al., 2008; Johnson and Halpern, 2012). On the other hand, neuropsychological studies in patients with lesions in the medial or lateral temporal lobes reveal dissociated recognition impairments for verbal and musical features of songs (Samson and Zatorre, 1991; Hébert and Peretz, 2001). These results suggest that the natural binding of lyrics and melodies into one unique song memory trace may be disrupted after brain damage. The present study seeks to find neural evidence for this hypothesis by investigating the effect of hippocampal damage on the emergence of integrated memory traces for lyrics and melodies during repeated exposure to songs.

Research over the last two decades testifies to a growing awareness that the hippocampus – beyond its classical role in explicit episodic memory (Scoville and Milner, 1957; Mishkin, 1982; Zola-Morgan and Squire, 1993) – plays a role in the implicit build-up of a memory trace (Chun and Phelps, 1999; Graham et al., 2010) and the bridging between perception and encoding (Bussey and Saksida, 2005; Baxter, 2009; Suzuki, 2009; Suzuki and Baxter, 2009; Olsen et al., 2012). According to the Emergent Memory Account (Graham et al., 2010) advancing a non-modular view of memory and perception, memory arises from a dynamic interaction between the perceptual representations distributed across the whole brain and a key role of the medial temporal lobe. More specifically, the hippocampus is thought to form conjunctive representations of inputs from unimodal and polymodal sensory cortices and to continuously return the processed information to the sensory cortex via feedback connections (McClelland et al., 1995; Eichenbaum, 2000; Turk-Browne et al., 2006; Bast, 2007), thus constantly updating the current representations with new experiences. This cortico-hippocampal loop of flowing information guarantees the encoding of events and its storage (Eichenbaum, 2000). Note that this mechanism not only implies a shared, anatomically distributed cerebral network for both memory and perception, but also puts the medial temporal lobe into a cardinal position between perceptual processes (Lee et al., 2005; Lee, 2006; Lee and Rudebeck, 2010a) and memory (long-term as well as short-term and working memory: Zarahn, 2004; Axmacher et al., 2007; Lee and Rudebeck, 2010b; Rose et al., 2012). Crucially, the hippocampus’ combined role in (i) memory formation and (ii) conjunction of sensory inputs (Sutherland and Rudy, 1989; Eichenbaum et al., 1994; Rudy and Sutherland, 1995; O’Reilly and Rudy, 2001; Winters, 2004; Cowell et al., 2006, 2010; Barense et al., 2007; Diana et al., 2007) makes it a potential key candidate for (i) the build-up of song memory traces, in which (ii) lyrics and melodies are integrated.

Although most of the studies on the hippocampus’ role in memory formation and binding come from the visual domain (Davachi, 2006; Diana et al., 2007; Shimamura, 2010), we hypothesize that similar processes also apply to the auditory domain (Overath et al., 2007, 2008; Buchsbaum and D’Esposito, 2009), especially to songs. It is reasonable to assume that memory formation for lyrics and melodies happens through a cortico-hippocampal loop, and that the natural combination of a verbal and a melodic component into a single song percept and memory trace requires binding mechanisms as described above. Tentative support for this comes from lesion studies in patients with anterior temporal lobectomy for treatment of pharmaco-resistant epilepsy (Samson and Zatorre, 1991). Using explicit recognition memory tasks after presentation of short unfamiliar songs, these experiments revealed a clear deficit in recognition of sung and spoken lyrics after left temporal lobe resection, and impaired recognition of melodies (without text) after right temporal lobe resection. On top of that, the data suggest a lack of integration of lyrics and melodies in patients with unilateral left (but not those with right) temporal lobe lesions. While patients with right temporal lobe resections had deficits in melody recognition when the tune was sung with new words, i.e., showing that they had bound the melody to the original lyrics, no such conjunction was observed in left-hemisphere damaged patients. In fact, their recognition of lyrics was impaired irrespective of whether these were presented with (or without) old or new melodies, suggesting an independent processing of the two song components and an isolated deficit for lyrics.

While these results lend initial support for our hypothesis of hippocampal involvement in song memory formation, they leave two important questions open: first, in how far can these deficit patterns be attributed to hippocampal dysfunctions, and second, in how far may these results depend on the use of a recognition memory task? First, the resection always included anterior temporal lobe structures beyond the hippocampus, making it difficult to pinpoint a specific hippocampal role. Furthermore, although the lesion description was based upon the surgeon’s meticulous drawings, a precise assessment of how far the resection extended into the hippocampus was not possible at that time. Moreover, although recognition tasks certainly depend on successful encoding, they also involve aspects of memory retrieval making it difficult to disentangle these effects with behavioral data. The present study seeks to address the points by first, testing patients with circumscribed unilateral hippocampal sclerosis (i.e., prior to surgery without further macroscopic lesions) and precisely describing the extent of hippocampal damage by means of volumetric analyses. Second, the incidental build-up of a song memory trace was assessed unbeknownst to the participants by examining the dynamics of neural adaptation during natural passive listening as described below.

Numerous studies have investigated the neural correlates of song processing (Samson and Zatorre, 1991; Brown et al., 2004a,b; Schön et al., 2005; Callan et al., 2006; Suarez et al., 2010; Merrill et al., 2012; Saito et al., 2012; Tierney et al., 2012), however, rarely has any study touched upon the implicit emergence of song memory. Indirect evidence can be drawn from studies using the successive presentation of changed and unchanged song stimuli (Same vs. Different) (Schön et al., 2010) and neural adaptation paradigms (Sammler et al., 2010). Adaptation is “a reduction of neural activity following prolonged or repetitive exposure to identical or at least similar stimuli” (Dobbins et al., 2004; Ganel et al., 2006; Grill-Spector et al., 2006), similar to repetition priming (Old vs. New stimuli) (Krekelberg et al., 2006). Although typically described in studies on perception, it appears that neural adaptation may also be indicative of memory trace formation. In line with the Emergent Memory Account (Graham et al., 2010), neural adaptation may reflect the emergence of a memory trace within cortical areas of perceptual representation through implicit learning during repeated exposure. Given the role of the hippocampus in memory formation (Turk-Browne et al., 2006) and according to connectionist models of memory (Damasio, 1989; McClelland et al., 1995; Rolls, 1996; Fuster, 1997), it is reasonable to suggest that cortical adaptation effects are subject to top-down modulations driven by the hippocampus (Blondin and Lepage, 2005; Goh et al., 2007), including integration of lyrics and melodies through binding (for a review on binding, see Opitz, 2010).

Of particular relevance for our research question of how lyrics and melody are bound in a conjunctive song memory trace are those studies describing the cerebral substrates underlying the integration of verbal and melodic components of songs (Sammler et al., 2010; Schön et al., 2010). These studies, which consider songs to be more than the sum of lyrics and melodies, examined modulations of brain activity to investigate how the two components interact, and how their processing is lateralized. For instance, Schön et al. (2010, Exp. 2) presented pairs of sung words that could vary or repeat in terms of the verbal and/or the melody component in a same-different task. Their results showed interactive processing in the left and the right superior temporal gyrus (STG), suggesting an integrated processing of the two components in these areas. Sammler et al. (2010) adopted a similar approach, taking advantage of neural adaptation effects. In this study, healthy participants were presented with blocks of short songs in which repetition of lyrics and/or melodies was varied in a factorial design to induce selective adaptation to lyrics, melodies, or unified songs. Consistent with Schön et al. (2010), repeated lyrics or repeated tunes evoked adaptation effects in bilateral STG. Core areas of integration were found in the left middle superior temporal sulcus (STS) and the left premotor cortex (PMC). Based on the previously reported literature, we hypothesize that these adaptation effects and the integration of lyrics and melodies are likely mediated by the hippocampus through feedback connections to STG/STS and binding of verbal and melodic information.

To investigate the modulatory effect of the hippocampus on (i) the incidental emergence of a song memory trace and (ii) the integration of the verbal and melodic components of songs, we adopted the paradigm by Sammler et al. (2010) to test patients with unilateral left or right hippocampal sclerosis and healthy controls. We compared the patterns of adaptation produced by songs in which either the lyrics, or the melodies, or both were repeated. As demonstrated by diffusion-weighted imaging studies, patients with hippocampal sclerosis present disconnections between medial and lateral temporal lobe regions (Focke et al., 2008; Bettus et al., 2009; Diehl et al., 2010; Riley et al., 2010; Liao et al., 2011). Such lesions have the capacity to prevent the hippocampus from sending feedback predictions and from updating the sensory memory trace (as expected by default after repetitions) and thus weaken adaptation effects in general and integration of lyrics and melodies in particular. More precisely, following Samson and Zatorre (1991), we hypothesized reduced adaptation for lyrics after left and for melodies after right hippocampal sclerosis. Moreover, following previous studies showing binding deficits in patients with left anterior temporal lobe resections (Samson and Zatorre, 1991) and correlates of lyrics–melody integration mainly in the left hemisphere (Sammler et al., 2010), we hypothesized that left hippocampal lesions, in particular, would have a negative impact on integration of lyrics and melodies in songs.

## Materials and Methods

### Participants

Twenty-four temporal lobe epilepsy patients with left (*n* = 12; LTLE) or right (*n* = 12; RTLE) hippocampal sclerosis participated in this study. They all presented with medically intractable epilepsy and were seen during pre-surgical evaluation at Pitié-Salpêtrière Hospital (Paris, France). All patients were right-handed according to the Edinburgh Handedness Inventory (Oldfield, 1971), except for one LTLE (−83.33) and one RTLE patient (−75). All patients had language lateralization to the left hemisphere except for the left-handed RTLE patient with bilateral language representation. Language lateralization was assessed by means of a verbal fluency test that is part of the standard functional magnetic resonance imaging (fMRI) assessment prior to epilepsy surgery at the Pitié-Salpêtrière Hospital. In the scanner, patients are required to think as many words of a semantic category (e.g., tools) as possible. The number of activated left and right fronto-temporo-parietal voxels against baseline was used to calculate a standard language lateralization score (Lehéricy et al., 2000; Thivard et al., 2005). The control group consisted of 19 right-handed healthy participants including 12 subjects, who had already participated in a previous study (Sammler et al., 2010), and 7 new volunteers. All participants were French native speakers and reported to have normal hearing. Controls were carefully selected to match the patient groups in terms of age, mean years of education, and musical expertise (Ehrlé musical expertise questionnaire, unpublished). A verbal memory deficit was present in the LTLE as opposed to the RTLE patients, as assessed with the Rey Auditory Verbal Learning Test (RAVLT) (Rey, 1964; Sziklas and Jones-Gotman, 2008) in accordance with the usual neuropsychological profile of these patients. Demographic characteristics of the participants are summarized in Table 1. The sclerosis in either left or right hippocampus in the two patient groups was corroborated by a volumetric analysis using Freesurfer software (Fischl, 2012; Reuter et al., 2012) that attested an ipsilateral hippocampal volume reduction of an average of 24.51% in the LTLE and 29.71% in the RTLE group compared to healthy controls. Between-group comparisons confirmed the significance of these volume reductions in the atrophic hippocampus (*p* < 0.05). Volumes and percentage of reduction are summarized in Table 2 (for details on the volumetric analysis, see Data Analysis). The local ethics committee approved this study and informed consent was obtained from each participant.

**Table 1:** **Demographic data**.

Group	*N*	Sex (males/females)	Mean age	Mean education	Musical expertise	Full scale IQ WAIS-R^a^	RAVLT forgetting%^a^
Control	19	9/10	32.63 ± 7.90	14.53 ± 2.99	5.02 ± 3.68	–	
LTLE	12	5/7	34.17 ± 8.71	12.25 ± 1.42	6.75 ± 4.46	92.66 ± 1.33	20.56 ± 22.99
RTLE	12	5/7	39.92 ± 1.23	12.67 ± 2.71	6.83 ± 5.45	95.43 ± 1.79	9.07 ± 10.76

*^a^ Mean for all except two RTLE patients due two missing data. RAVLT: Rey Auditory Verbal Learning Test*.

**Table 2:** **Medial temporal lobe (MTL) volumes (mm^3^)**.

	Left MTL				Right MTL			
	LTLE		RTLE		LTLE		RTLE	
Region	Mean/ ± SD	Reduction (%)^a^	Mean/ ± SD	Reduction (%)^a^	Mean/ ± SD	Reduction (%)^a^	Mean/ ± SD	Reduction (%)^a^
Hippocampus	2606.17/506.82	24.51	3467.95/245.68	−0.46	3589.04/678.97	−2.72	2455.90/414.39	29.71
Entorhinal cortex	1802.67/613.03	3.86	1847.25/218.17	1.48	1901.42/408.28	3.73	1802/246.24	8.76
Parahippocampal gyrus	2165.42/386.72	8.56	2304.17/222.16	2.70	2249.92/266.37	3.26	2026/268.47	12.89

*^a^ Percentage of reduced volumes as compared to control group volumes*.

### Materials

The material and the scanning protocol used here were previously published by Sammler et al. (2010). The stimulus set consisted of 48 blocks of 6 unfamiliar songs based on a collection of nineteenth century French folk songs (Robine, 1994). Each song within a block was sung by a different singer to avoid adaptation to the singer’s voice (Belin and Zatorre, 2003), had a duration of 2.5 s and was followed by a 0.2 s pause. Repetition of lyrics and/or melodies within blocks was crossed in a 2 × 2 factorial design, forming four conditions. Songs within a block either had the same melodies and same lyrics (S_M_S_L_), the same melodies but different lyrics (S_M_D_L_), different melodies with same lyrics(D_M_S_L_), or different melodies and different lyrics (D_M_D_L_). Mode and tempo were balanced across the stimulus set, and each song had an average of 7.65 notes and 5.61 words. Songs in the four conditions did not differ with respect to length and number of word/note, word frequency, interval size, and number of contour reversals. In blocks where lyrics were varied, they did not rhyme, were semantically distant, and differed with respect to syntactic structure avoiding potential adaptation to phonology, semantic content, or syntactic structure (Noppeney and Price, 2004).

### Procedure

Participants were instructed to listen attentively with closed eyes while avoiding moving, humming, or singing along. No behavioral data were collected. Stimuli were presented using E-Prime 1.1 (Psychology Software Tools) and delivered binaurally through air pressure headphones (MR confon). Additionally, participants used earplugs to minimize noise interference. All blocks were presented in one of four pseudorandom orders, with a silent gap between blocks of 10 s (±0.5 s) allowing the hemodynamic response to return to baseline (Belin and Zatorre, 2003). This resulted in a total duration of the experiment of around 30 min. Blocks of the same condition were not presented more than twice in a row. At the end of the experiment, all participants filled in a debriefing questionnaire with several nine-point scales (1 = not at all, 9 = always) in which they rated their attention during listening at 7.63 (Controls), 7.00 (LTLE), 7.57 (RTLE), and the amount of overt and/or covert singing during scanning at 0.00 and 2.89 (Controls), 0.47 and 2.71 (LTLE), and 0.21 and 2.14 (RTLE), showing that they had followed the instructions.

### Scanning

Functional magnetic resonance imaging was performed using a 3-T Siemens TRIO scanner (Siemens, Erlangen, Germany) at the *Centre de Neuroimagerie de Recherche* at the *Institut du Cerveau et de la Moëlle Épinière – ICM* (Groupe Hospitalier Pitié-Salpêtrière, Paris, France). Radiofrequency transmission was performed with a body coil and the signal was received with a 12-channel head coil. Before the functional scans, high-resolution T1-weighted images (1 × 1 × 1 mm^3^ voxel size) were collected for anatomical coregistration using a magnetization-prepared rapid acquisition gradient-echo (MPRAGE) sequence (TR = 2300 ms, TE = 4.18 ms). Subsequently, one series of 595 blood oxygenation level-dependent (BOLD) images was obtained using a single-shot echo-planar gradient-echo (EPI) pulse sequence (TR = 2120 ms, TE = 25 ms, the first six volumes were later discarded to allow for T1 saturation). Forty-four interleaved slices (3 mm × 3 mm × 3 mm voxel size, 10% interslice gap) perpendicular with respect to the hippocampal plane were collected. The field of view was 192 × 192 mm^2^ with an in-plane resolution of 64 × 64 pixels and a flip angle of 90°. Scanner noise was continuous during the experiment representing a constant auditory background.

### Data analysis

The fMRI data were analyzed using SPM8 (Wellcome Trust Centre for Neuroimaging). Preprocessing included spatial realignment and reslicing and coregistration of the anatomical T1 to the mean functional data. The first level analysis was carried out in the native space. Four regressors were built for each experimental condition based on the general linear model (different melodies and different lyrics (D_M_D_L_); same melodies and different lyrics (S_M_D_L_); different melodies and same lyrics (D_M_S_L_) and same melodies and same lyrics (S_M_S_L_), and convolved with a hemodynamic response function (HRF). Movement parameters were included as regressors of no interest and serial correlations were modeled with an AR (1) process. A temporal high-pass filter with a cut-off of 200 s was used to eliminate low-frequency drifts. Six one-sample *t*-tests were computed for each participant: all conditions against silence to establish a “song-sensitive” mask, the main effects of adaptation to lyrics [(D_M_D_L_ + S_M_D_L_) – (D_M_S_L_ + S_M_S_L_)] and to melodies [(D_M_D_L_ + D_M_S_L_) – (S_M_D_L_ + S_M_S_L_)] to identify areas of general adaptation to the repetition of song components, as well as the interaction [(D_M_S_L_ + S_M_D_L_) – (D_M_D_L_ + S_M_S_L_)] to isolate areas of lyrics–melody integration. For the sake of completeness and consistency with the analysis of Sammler et al. (2010), we additionally compared both main effects to identify brain regions that showed an independent processing of either lyrics or melodies (i.e., stronger adaptation for lyrics than for melodies [2 × (S_M_D_L_)] and vice versa [2 × (D_M_S_L_)]).

Segmentation of the anatomical files was performed with the VBM8 toolbox (Ashburner and Friston, 2005) to form a normalized anatomical image and the DARTEL exported tissue types. A template with eight iterations was created in DARTEL (Ashburner, 2007) including all 43 subjects to improve anatomical accuracy in the normalization of the functional contrast images obtained in the first level. Contrast images were spatially smoothed using a three-dimensional Gaussian kernel with 8 mm full width at half maximum. For the second level, the DARTEL normalized contrast images were normalized to the Montreal Neurological Institute (MNI) space. The automatically generated mask from the first level analysis of each subject was also normalized with this procedure but without smoothing. Statistical analysis was confined to a song-sensitive mask in gray matter to increase signal detection (Friston et al., 1994). To create this mask, a binary mask from the last iteration of the DARTEL template thresholded at 0.3 was overlaid with active voxels in the “all conditions against silence” contrast at *p* < 0.05 (FWE correction for multiple comparisons), *k* > 5 for all 43 participants. All voxels that were involved in both were included into the explicit song-sensitive mask for statistics. This mask covered an auditory-motor network, including the temporal gyrus, the PMC, and the cerebellum. For random effects group analyses, the individual contrast images were submitted to one-sample *t*-tests, separately for healthy controls, LTLE and RTLE patients. Furthermore, two-sample *t*-tests were computed for all contrasts, comparing each patient group against controls. All SPMs were threshold at *p* < 0.001 (uncorrected) with a minimum cluster extent of *k* ≥ 5 voxels. Results will report the peak voxel *p* value and the number of voxels (*k*).

To assess the size of the hippocampal sclerosis and surrounding cortex, volumetric measures of hippocampal, entorhinal, and parahippocampal gyrus were obtained for all participants with the Freesurfer image analysis suite (Fischl, 2012; Reuter et al., 2012), which is documented and freely available for downloading online (http://surfer.nmr.mgh.harvard.edu/). Non-parametric tests (Kruskal–Wallis, SPSS 18.0) were used to compare these measures between the patient and controls groups. To control global differences, intracranial volume was included in the analysis as a covariate, which was not found to be significant. The percentage of reduction of each structure was calculated for each patient group in comparison to the control group and is reported in Table 2.

## Results

### Main effects

**Figure 1:**
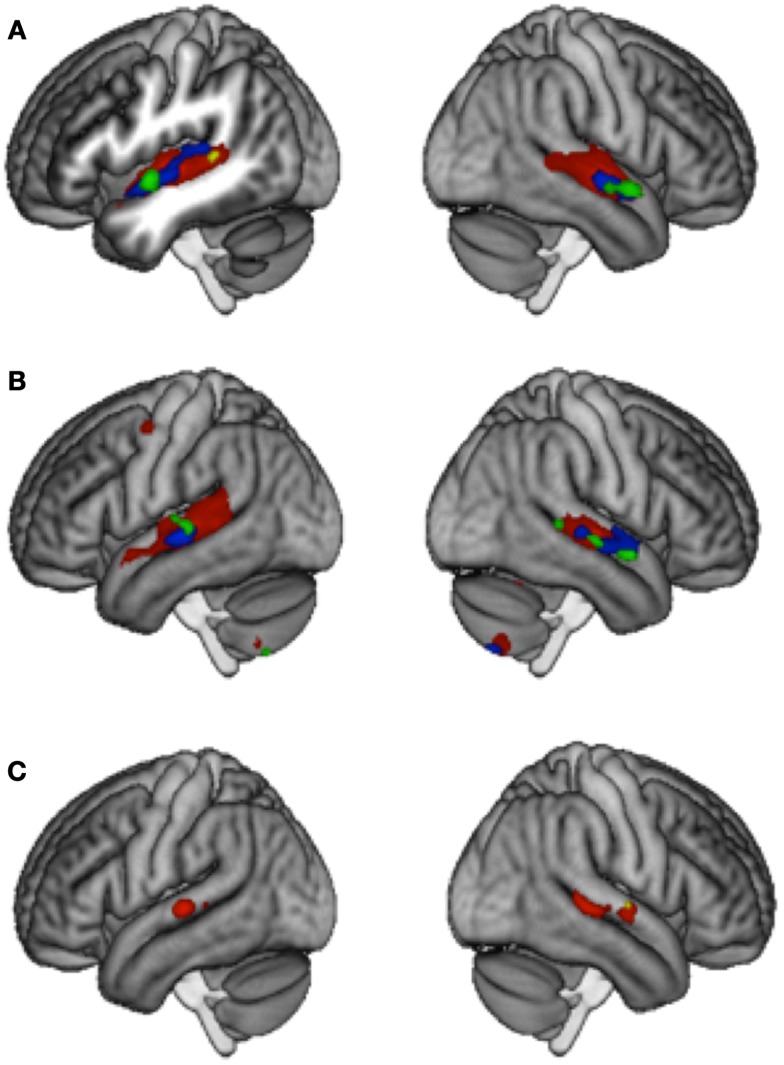
**Main effects of Adaptation to Lyrics (A) and Melody (B), and the Interaction (integration contrast) (C)**. Threshold *p * < 0.001 *k* ≥ 5 uncorrected. Results for Control group (red), LTLE (blue), RTLE (green), and Controls vs. LTLE (yellow).

A complete report of the results at threshold *p* < 0.001 (uncorrected) with a minimum cluster extent of *k* ≥ 5 voxels can be seen in Table 3. All three groups of participants showed adaptation to lyrics in the left and right STG and STS that was however considerably more extended in Controls (2474 and 2423 voxels) than in LTLE (541 and 388 voxels) and RTLE patients (201 and 165 voxels). Between-group comparisons revealed significantly weaker adaptation effects in the LTLE but not in the RTLE as compared to Controls in the left STS (Figure 1A).

**Table 3:** **Main effects of lyrics and melodies repetition for each group and comparison between Controls and LTLE**.

Group	Adaptation for lyrics				Adaptation for melody			
	Area	Size(*k*)	*x*, *y*, *z* (mm)	*Z*	Area	Size(*k*)	*x*, *y*, *z* (mm)	*Z*
Control	Left hemisphere				Left hemisphere			
	STG/STS	2474	−58, −6, −6	5.75	STG/STS	2380	−64, −29, 3	5.12
			−62, −17, 0	5.52			−54, −39, 3	4.75
			−48, −39, 6	5.04			−66, −39, 12	4.68
	Temporal pole	11	−51, 9, −18	3.86	PrCG	52	−52, −5, 51	3.94
					Cerebellum	55	−26, −62, −55	3.75
	Right hemisphere				Right hemisphere			
	STG/STS	2423	62, −9, −6	5.59	STG/STS	1830	60, −17, −3	5.85
			60, −0, −13	4.76			46, −36, 2	4.26
			62, −24, 2	4.39			62, −0, −10	4.12
	Cerebellum	10	16, −80, −46	3.26	Cerebellum	148	34, −63, −58	3.91
					Cerebellum	27	30, −57, −27	3.59
LTLE	Left hemisphere				Left hemisphere			
	STG/STS	541	−58, −5, −9	4.60	STG	245	−57, −21, −3	4.68
			−56, −15, −3	4.05				
			−66, −33, 9	3.65				
	Right hemisphere				Right hemisphere			
	STG/STS	388	62, −9, −7	4.41	STG/STS	295	62, 2, −9	3.69
			62, 2, −13	3.85			57, −11, −9	3.67
							58, −5, −1	3.44
					STG/STS	92	54, −24, 0	3.69
					Cerebellum	134	26, −74, −60	3.67
RTLE	Left hemisphere				Left hemisphere			
	STG/STS	201	−63, −6, −6	4.26	STG/STS	106	−66, −26, 2	3.55
							−58, −18, 6	3.24
					Cerebellum	20	−24, −66, −60	3.30
	Right hemisphere				Right hemisphere			
	Temporal pole	165	62, 3, −10	4.13	STG/STS	61	62, −0, −12	3.95
	STG/STS		63, −9, −10	3.29	STG/STS	50	58, −17, −4	3.65
					STG/STS	21	69, −36, 3	3.41
Control vs. LTLE	Left hemisphere				Left hemisphere			
	STS	25	−46, −39, 6	3.60				

In all three groups, adaptation to melody was found in the left and right STG and STS, again more extended in Controls (2380 and 1830 voxels) than in LTLE (245 and 295 voxels) and RTLE patients (106 and 111 voxels), as well as in the cerebellum. The Control group showed, in addition, adaptation in the left PMC (52 voxels) that was not observed in patients (Figure 1B). However, between-group differences failed to reach significance.

### Interaction effects

**Figure 2:**
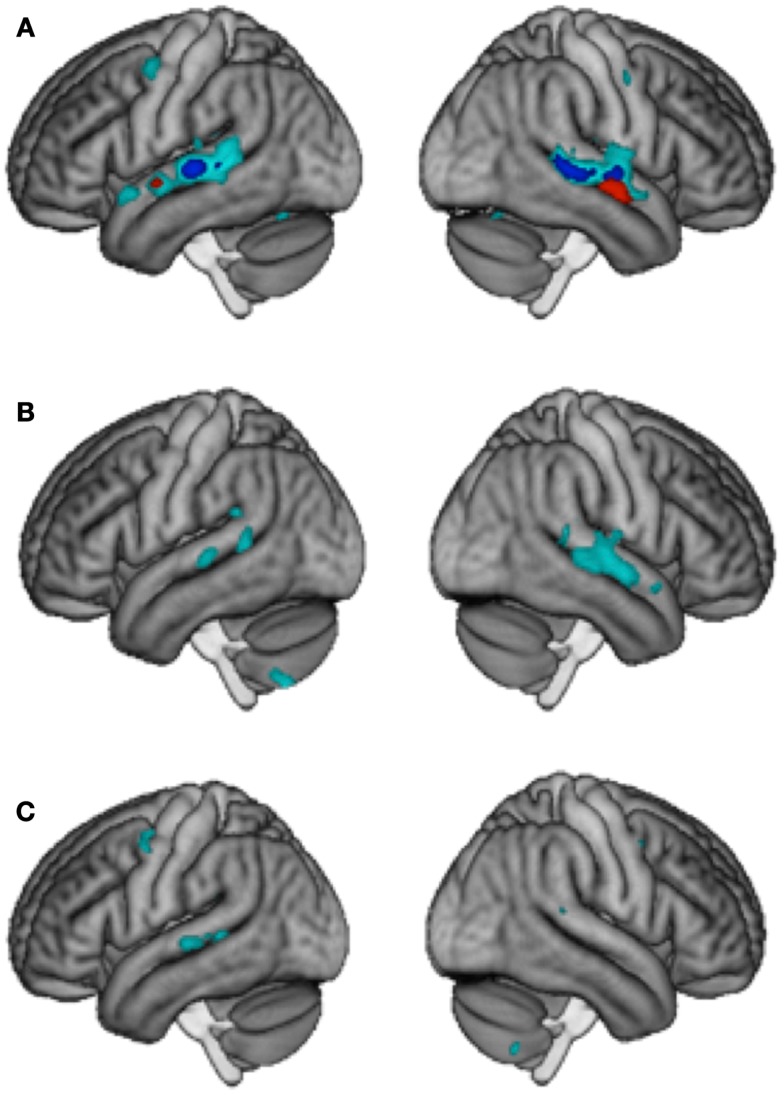
**“Gradient of integration” for (A) the Control group (B) RTLE and (C) LTLE patients**. Specificity for lyrics is shown in red (*p* < 0.001 *k* > 5 uncorr.), interaction in dark blue (*p* < 0.001 *k* > 5 uncorr.) and weaker interaction in cyan (Interaction at *p* < 0.05 *k* > 5 uncorr.).

Interaction effects were calculated with the contrast [(D_M_S_L_ + S_M_D_L_) – (D_M_D_L_ + S_M_S_L_)] and were taken to represent an integrated processing of lyrics and melodies in songs. Only the control group showed interaction effects at *p* < 0.001 *k* ≥ 5, which were located in the bilateral posterior STG/STS (left: 169 voxels and right: 323 voxels). No such effect was observed in LTLE and RTLE patients. To visualize areas that simply may not have passed our statistical criterion, we inspected the data at a very lenient level of *p* < 0.05 uncorrected (*k* > 5). Controls showed an extended region within the left (1936 voxels) and right (2176 voxels) STG/STS (Figure 2A). At this threshold, RTLE patients showed a pattern that was similar to Controls, but considerably less extended (554 and 1501 voxels) (Figure 2B). Interestingly, LTLE patients showed nearly no interaction in the temporal lobe at this very lenient threshold (238 and 35 voxels) (Figure 2C). Indeed, between-group comparisons revealed a significantly weaker interaction effect in the LTLE than the Control group in the right STG (Figure 1C) whereas the difference between the RTLE patients and Controls did not reach significance. Details on interaction effects are shown in Table 4.

**Table 4:** **Integration and independence for each group and between controls and LTLE**.

**INTEGRATION**				
Group	Area	Size (*k*)	*x*, *y*, *z* (mm)	*Z*
Control	Left hemisphere
	STG	164	−63, −23, 2	4.39
	MTG	5	−63, −36, 3	3.18
	Right hemisphere
	STG	235	56, −32, 0	3.98
			51, −38, 5	3.65
	STG	82	66, −11, −3	3.76
	STG	6	66, −20, 0	3.18
Control vs. LTLE	Right hemisphere
	STG	6	63, −9, 0	3.19
**LYRICS INDEPENDENCE**				
Control	Left hemisphere
	STG	15	−63, −5, −6	3.29
	STG	8	−46, −41, 8	3.32
	Right hemisphere
	STG	196	63, −6, −15	3.79
**MELODY INDEPENDENCE**				
LTLE	Left hemisphere
	Cerebellum	10	−18, −66, −61	3.58
RTLE	Left hemisphere
	Cerebellum	19	−12, −83, −46	3.77
	Cerebellum	6	−24, −66, −61	3.21
	Right hemisphere
	Cerebellum	18	14, −81, −46	3.62

### Independence effects

Greater adaptation to lyrics as compared to melody was found bilaterally in the anterior region of the STG (23 and 196 voxels) in the control group, suggesting an independent processing of lyrics in this region. Greater adaptation to melody as compared to lyrics was obtained bilaterally in the cerebellum in RTLE patients. However, between-group differences failed to reach significance (Figure 2A). Details on independence effects are shown in Table 4.

## Discussion

The aim of the current study was to assess the modulatory effects of a unilateral hippocampal lesion on the incidental emergence of a song memory trace and the integration of lyrics and melodies into a conjunctive representation. To this end, neural adaptation to song repetition – as a proxy for song memory formation – was examined in patients with left or right hippocampal sclerosis in comparison to healthy controls using an fMR-adaptation paradigm. It was hypothesized that damage to the hippocampus may disrupt feedback connections to the lateral temporal lobe and thus preclude the establishment and update of a sensory memory trace. As a consequence, damage to the hippocampus may result in weaker neural adaptation in the STG. In particular, hippocampal lesions could hinder the integration of lyrics and melodies into a unified memory trace (Diana et al., 2007; Staresina and Davachi, 2009; Graham et al., 2010; Shimamura, 2010).

The main findings of this study were indeed that the neural adaptation to lyrics repetition as well as the integration of lyrics and melodies in songs (as reflected by the statistical interaction between adaptation effects for lyrics and melodies) was reduced in patients with left hippocampal sclerosis. More specifically, the direct comparison of these patients with healthy control participants revealed a weaker adaptation to lyrics in the left STS and a weaker integration of lyrics and melodies in the right STG. If one accepts the notion that neural adaptation reflects the emergence of a memory trace (see Introduction), these results are in line with our hypotheses and previous work showing that left hippocampal damage may lead to weaker memory for lyrics (Samson and Zatorre, 1991) and may hinder the integration of lyrics and melodies into a unified memory representation (Samson and Zatorre, 1991; Sammler et al., 2010).

All three groups of participants showed adaptation to the repetition of lyrics or melodies in the bilateral STG and STS, but in both patient groups, these effects were markedly smaller in spatial extent when compared to healthy controls. Notably, patients with left (but not right) hippocampal sclerosis exhibited significantly decreased adaptation to lyrics in the left STS, which is known to play a role in phonemic processing and also known to be crucial for the perception of a sound as speech (Dehaene-Lambertz et al., 2005; Liebenthal, 2005; Möttönen et al., 2006; for a review on STS, see Hein and Knight, 2008). This finding is most likely tied to the role of the left medial temporal lobe in verbal processing (Meyer et al., 2005; Wagner et al., 2008; Greve et al., 2011) and may reflect the perturbed build-up of memory traces for lyrics (and verbal material in general) due to disrupted feedback connections between medial and lateral structures of the left temporal lobe (Eichenbaum, 2000). Such an interpretation could be supported by the verbal memory deficit documented in the LTLE patients of the present study (assessed with the RAVLT) and, although we did not collect behavioral data for this experiment, these results are also in agreement with the behavioral results of Samson and Zatorre (1991). That study showed that the recognition of sung lyrics after listening to unfamiliar songs was impaired in patients with left (but not right) medial temporal lobe lesions.

Although patients with right hippocampal sclerosis showed nominally reduced adaptation and integration effects, these did not significantly differ from those in healthy controls, suggesting rather normal song processing and lyrics–melody integration in these patients. While the latter is in line with previous behavioral data showing spared integration of lyrics and tunes after right anterior temporal lobe resection (Samson and Zatorre, 1991), our hypothesis on reduced adaptation to melodies was not confirmed. This may partly be due to the stimulus material used: even if melodies were repeated to induce adaptation, they differed in octave sung by sopranos, tenors, altos, and bass. Most likely, adaptation effects are not fully robust to transposition of melodies. Furthermore, adaptation to melodies was generally weaker than adaptation to lyrics, as attested by the results in healthy participants, possibly resulting in a floor effect. Our participants may have paid less attention to melodies than to lyrics (as the latter convey the message) leading to weak adaptation, given that a lack of attention reduces adaptation effects (Chee and Tan, 2007). Alternatively, several lines of evidence suggest that melodies may be processed more bilaterally than lyrics (Samson and Zatorre, 1992; Binder et al., 2000; Besson and Schön, 2003; Peretz and Coltheart, 2003; Schön et al., 2005; Patel, 2008; Koelsch, 2012), leading to less severe deficits in processing melodies than in verbal processing after unilateral temporal lobe damage. Further studies will be necessary to clarify this issue.

One novel finding is the main effect of melodies in the cerebellum in all groups (without group differences). Since activity in the cerebellum has been frequently reported in other studies using sung material (Parsons, 2001; Callan et al., 2007; Lebrun-Guillaud et al., 2008; Tillmann et al., 2008; Merrill et al., 2012), these effects may be linked to optimization of the fine sensory acquisition and internalization of input–output characteristics of stimuli, a process related to the creation of internal models of vocal articulation (Parsons, 2001; Callan et al., 2007; Stoodley and Schmahmann, 2009), that may function independently from the hippocampus.

As previously reported (Sammler et al., 2010), healthy participants presented maximum integration of lyrics and melodies in the posterior STS with a continuous decay of the lyrics–melodies integration along the posterior–anterior axis, toward regions of independent processing of lyrics in the anterior STG. These effects were shown bilaterally in the present experiment, expanding the previously reported effect, which was restricted to the left hemisphere. This analysis illustrates a “gradient of integration” from more to less integrated processing. In line with the literature on music and language (Scott et al., 2000; Davis and Johnsrude, 2003; Scott and Johnsrude, 2003; Friederici, 2011; Gow, 2012), this gradient poses an integrative processing of songs at the prelexical and phonemic level in the mid-STS. Consequently, information can be transmitted both along an anterior pathway to the temporal pole for an independent analysis of the linguistic content, and along a posterior pathway to the left PMC for the integrated sensori-motor conversion of the stimuli. In other words, lyrics and melodies might split up in the ventral pathway for semantics and comprehension (Griffiths, 2001; Patterson et al., 2002; Hickok and Poeppel, 2007; Saur et al., 2008; Friederici, 2009, 2011; Hickok et al., 2011) but stay integrated in sensori-motor dorsal pathways (Kiebel et al., 2008; Loui et al., 2009).

Contrary to healthy participants, both patient groups showed very weak levels of lyrics–melody integration in the bilateral mid-STG/STS, and only after lowering the statistical threshold to *p* < 0.05 (uncorrected). This effect may reside on generally weaker adaptation effects in both patient groups. The spatial extent of this weak lyrics–melody interaction was particularly small in patients with left hippocampal sclerosis who also showed a significantly reduced interaction effect in the right STG as compared to controls. These tendencies suggest a partial (although not complete) disruption of integrated processing in clinical populations and indicate that the conjunctive representation of lyrics and melodies depends on intact medial temporal lobe structures, particularly in the left hemisphere. Overall, this finding is in line with previous studies in patients with anterior temporal lobe resection including parts of the hippocampus (Samson and Zatorre, 1991). These experiments showed a perturbed integration of verbal and melodic song components in patients with left (but not right) temporal lobe resections, i.e., a selective deficit in recognizing lyrics that was independent from recognition memory for melodies. It is worth to mention that in both the present and previous studies, the integration deficit may reside on a more general deficit to process lyrics, as supported by the weaker adaptation for lyrics and reduced performance in neuropsychological tests on verbal memory in our patients with left hippocampal sclerosis.

Taken together, adaptation to lyrics and integration of lyrics and melodies within songs appear to be less efficient in patients with left hippocampal damage as compared to healthy controls. We propose that these lesions may hinder the build-up of a sensory memory trace for lyrics (with rather preserved mechanisms for melodies), which in turn might be at the origin of the reduced integration of lyrics and melody. These combined effects could be attributed to hippocampal malfunction *per se* or to a more global disconnection of lateral temporal neocortical structures caused by repetitive seizures or epilepsy history (Yasuda et al., 2010; Besson et al., 2012), both of which can disrupt the hippocampal top-down modulatory influence on STG/STS. If this is the case, it is possible that adaptation could also be reduced for stimuli other than lyrics, melodies, or songs, demonstrating a more general adaptation and putative encoding deficit following disruption of cortico-hippocampal processing loops.

Interestingly, an independent analysis of the connectivity profiles in our patients showed asymmetries between the left and right hemispheric lesion groups: LTLE patients exhibited more extended and more strongly left-lateralized disconnections, as opposed to more discrete and bilateral connectivity deficits in RTLE (Besson et al., 2012). Such differences in connectivity profiles provide an additional explanation for the nominally stronger impairments in patients with left hippocampal sclerosis as compared to patients with right hippocampal sclerosis. In sum, the present data indicate that an imbalance in the left hippocampo-cortical system, due to hippocampal sclerosis and/or disrupted connectivity with STG/STS, affects the incidental emergence of a memory trace of verbal song components and precludes the build-up of a conjunctive representation that integrates lyrics and melodies.

## Conclusion

To the best of our knowledge, this is the first study to investigate the processing of songs using fMRI in patients with unilateral hippocampal sclerosis. We showed that the adaptation to lyrics and the integration of lyrics and melodies was diminished in lateral temporal lobe regions in patients with left hippocampal sclerosis while a similar but non-significant result pattern was found in patients with right hippocampal sclerosis. These findings suggest the importance of hippocampal top-down modulations on the STG/STS during repetitive exposure to songs. We interpret the observed adaptation patterns to be a result of a disturbed connectivity in a hippocampal–cortical network, weakening the emergence of a memory trace for lyrics and the integrated processing of songs as a unified percept. Overall, these data provide a novel contribution by suggesting that the integration shown in healthy participants is tied to the integrity of the medial temporal lobe and its connections with the lateral temporal cortex.

## Conflict of Interest Statement

The authors declare that the research was conducted in the absence of any commercial or financial relationships that could be construed as a potential conflict of interest.

## References

[B1] Ashburner J. (2007). A fast diffeomorphic image registration algorithm. Neuroimage 38, 95–113 10.1016/j.neuroimage.2007.07.007 17761438

[B2] Ashburner J. Friston K. J. (2005). Unified segmentation. Neuroimage 26, 839–851 10.1016/j.neuroimage.2005.02.018 15955494

[B3] Axmacher N. Mormann F. Fernandez G. Cohen M. X. Elger C. E. Fell J. (2007). Sustained neural activity patterns during working memory in the human medial temporal lobe. J. Neurosci. 27, 7807–7816 10.1523/JNEUROSCI.0962-07.2007 17634374 PMC6672876

[B4] Barense M. D. Gaffan D. Graham K. S. (2007). The human medial temporal lobe processes online representations of complex objects. Neuropsychologia 45, 2963–2974 10.1016/j.neuropsychologia.2007.05.023 17658561

[B5] Bast T. (2007). Toward an integrative perspective on hippocampal function: from the rapid encoding of experience to adaptive behavior. Rev. Neurosci. 18, 253–282 10.1515/REVNEURO.2007.18.3-4.253 18019609

[B6] Baur B. Uttner I. Ilmberger J. Fesl G. Mai N. (2000). Music memory provides access to verbal knowledge in a patient with global amnesia. Neurocase 6, 415–421 10.1080/13554790008402712

[B7] Baxter M. G. (2009). Involvement of medial temporal lobe structures in memory and perception. Neuron 61, 667–677 10.1016/j.neuron.2009.02.007 19285463

[B8] Belin P. Zatorre R. (2003). Adaptation to speaker’s voice in right anterior temporal lobe. Brain Imaging 14, 2105–2109 10.1097/01.wnr.0000091689.94870.85 14600506

[B9] Besson M. Schön D. (2003). Comparison between language and music. Ann. N. Y. Acad. Sci. 930, 232–258 10.1111/j.1749-6632.2001.tb05736.x 11458832

[B10] Besson P. Dinkelacker V. Valabregue R. Samson S. Thivard L. Baulac M. (2012). “Decreased connectivity of major cortical nodes is more pronounced and lateralized in left versus right mesial temporal sclerosis,” in Poster Presented at 66th American Epilepsy Society Annual Meeting, San Diego

[B11] Bettus G. Guedj E. Joyeux F. Confort-Gouny S. Soulier E. Laguitton V. (2009). Decreased basal fMRI functional connectivity in epileptogenic networks and contralateral compensatory mechanisms. Hum. Brain Mapp. 30, 1580–1591 10.1002/hbm.20625 18661506 PMC6870867

[B12] Binder J. R. Frost J. A. Hammeke T. Bellgowan P. S. Springer J. Kaufman J. (2000). Human temporal lobe activation by speech and nonspeech sounds. Cereb. Cortex 10, 512–528 10.1093/cercor/10.5.512 10847601

[B13] Blondin F. Lepage M. (2005). Decrease and increase in brain activity during visual perceptual priming: an fMRI study on similar but perceptually different complex visual scenes. Neuropsychologia 43, 1887–1900 10.1016/j.neuropsychologia.2005.03.021 16168731

[B14] Brown S. Martinez M. J. Hodges D. A. Fox P. T. Parsons L. M. (2004a). The song system of the human brain. Brain Res. Cogn. Brain Res. 20, 363–375 10.1016/j.cogbrainres.2004.03.016 15268914

[B15] Brown S. Martinez M. J. Parsons L. M. (2004b). Passive music listening spontaneously engages limbic and paralimbic systems. Neuroreport 15, 2033–2037 10.1097/00001756-200409150-00008 15486477

[B16] Buchsbaum B. R. D’Esposito M. (2009). Repetition suppression and reactivation in auditory-verbal short-term recognition memory. Cereb. Cortex 19, 1474–1485 10.1093/cercor/bhn186 18987393 PMC2677654

[B17] Bussey T. J. Saksida L. M. (2005). Object memory and perception in the medial temporal lobe: an alternative approach. Curr. Opin. Neurobiol. 15, 730–737 10.1016/j.conb.2005.10.014 16271459

[B18] Callan D. E. Kawato M. Parsons L. Turner R. (2007). Speech and song: the role of the cerebellum. Cerebellum 6, 321–327 10.1080/14734220601187733 17853077

[B19] Callan D. E. Tsytsarev V. Hanakawa T. Callan A. M. Katsuhara M. Fukuyama H. (2006). Song and speech: brain regions involved with perception and covert production. Neuroimage 31, 1327–1342 10.1016/j.neuroimage.2006.01.036 16546406

[B20] Chee M. W. Tan J. C. (2007). Inter-relationships between attention, activation, fMR adaptation and long-term memory. Neuroimage 37, 1487–1495 10.1016/j.neuroimage.2007.07.006 17689983

[B21] Chun M. M. Phelps E. A. (1999). Memory deficits for implicit contextual information in amnesic subjects with hippocampal damage. Nat. Neurosci. 2, 844–847 10.1038/12222 10461225

[B22] Cowell R. A. Bussey T. J. Saksida L. M. (2006). Why does brain damage impair memory? A connectionist model of object recognition memory in perirhinal cortex. J. Neurosci. 26, 12186–12197 10.1523/JNEUROSCI.2818-06.2006 17122043 PMC6675420

[B23] Cowell R. A. Bussey T. J. Saksida L. M. (2010). Components of recognition memory: dissociable cognitive processes or just differences in representational complexity? Hippocampus 20, 1245–1262 10.1002/hipo.20865 20882548

[B24] Crowder R. G. Serafine M. L. Repp B. (1990). Physical interaction and association by contiguity in memory for the words and melodies of songs. Mem. Cognit. 18, 469–476 10.3758/BF03198480 2233260

[B25] Damasio A. R. (1989). The brain binds entities and events by multiregional activation from convergence zones. Neural Comput. 1, 123–132 10.1162/neco.1989.1.1.123

[B26] Davachi L. (2006). Item, context and relational episodic encoding in humans. Curr. Opin. Neurobiol. 16, 693–700 10.1016/j.conb.2006.10.012 17097284

[B27] Davis M. H. Johnsrude I. S. (2003). Hierarchical processing in spoken language comprehension. J. Neurosci. 23, 3423–3431 12716950 10.1523/JNEUROSCI.23-08-03423.2003 PMC6742313

[B28] Dehaene-Lambertz G. Pallier C. Serniclaes W. Sprenger-Charolles L. Jobert A. Dehaene S. (2005). Neural correlates of switching from auditory to speech perception. Neuroimage 24, 21–33 10.1016/j.neuroimage.2004.09.039 15588593

[B29] Diana R. A. Yonelinas A. P. Ranganath C. (2007). Imaging recollection and familiarity in the medial temporal lobe: a three-component model. Trends Cogn. Sci. (Regul. Ed.) 11, 379–386 10.1016/j.tics.2007.08.001 17707683

[B30] Diehl B. Tkach J. Piao Z. Ruggieri P. LaPresto E. Liu P. (2010). Diffusion tensor imaging in patients with focal epilepsy due to cortical dysplasia in the temporo-occipital region: electro-clinico-pathological correlations. Epilepsy Res. 90, 178–187 10.1016/j.eplepsyres.2010.03.006 20542410

[B31] Dobbins I. G. Schnyer D. M. Verfaellie M. Schacter D. L. (2004). Cortical activity reductions during repetition priming can result from rapid response learning. Nature 428, 316–319 10.1038/nature02400 14990968

[B32] Eichenbaum H. (2000). A cortical-hippocampal system for declarative memory. Nat. Rev. Neurosci. 1, 41–50 10.1038/35036213 11252767

[B33] Eichenbaum H. Otto T. Cohen N. J. (1994). Two functional components of the hippocampal memory system. Behav. Brain Sci. 17, 449–471 10.1017/S0140525X00035391

[B34] Fischl B. (2012). FreeSurfer. Neuroimage 62, 774–781 10.1016/j.neuroimage.2012.01.021 22248573 PMC3685476

[B35] Focke N. K. Yogarajah M. Bonelli S. B. Bartlett P. A. Symms M. R. Duncan J. S. (2008). Voxel-based diffusion tensor imaging in patients with mesial temporal lobe epilepsy and hippocampal sclerosis. Neuroimage 40, 728–737 10.1016/j.neuroimage.2007.12.031 18261930

[B36] Friederici A. D. (2009). Pathways to language: fiber tracts in the human brain. Trends Cogn. Sci. (Regul. Ed.) 13, 175–181 10.1016/j.tics.2009.01.001 19223226

[B37] Friederici A. D. (2011). The brain basis of language processing: from structure to function. Physiol. Rev. 91, 1357–1392 10.1152/physrev.00006.2011 22013214

[B38] Friston K. Jezzard P. Turner R. (1994). Analysis of functional MRI time-series. Hum. Brain Mapp. 1, 153–171 10.1002/hbm.460010207

[B39] Fuster J. M. (1997). Network memory. Trends Neurosci. 20, 451–459 10.1016/S0166-2236(97)01128-4 9347612

[B40] Ganel T. Gonzalez C. L. Valyear K. F. Culham J. C. Goodale M. A. Köhler S. (2006). The relationship between fMRI adaptation and repetition priming. Neuroimage 32, 1432–1440 10.1016/j.neuroimage.2006.05.039 16854597

[B41] Goh J. O. S. Chee M. W. L. Tan J. Venkatraman V. Hebrank A. Leshikar E. (2007). Age and culture modulate object processing and object-scene binding in the ventral visual areas. Cogn. Affect. Behav. Neurosci. 7, 44–52 10.3758/CABN.7.1.44 17598734

[B42] Gow D. W. (2012). The cortical organization of lexical knowledge: a dual lexicon model of spoken language processing. Brain Lang. 121, 273–288 10.1016/j.bandl.2012.03.005 22498237 PMC3348354

[B43] Graham K. S. Barense M. D. Lee A. C. (2010). Going beyond LTM in the MTL: a synthesis of neuropsychological and neuroimaging findings on the role of the medial temporal lobe in memory and perception. Neuropsychologia 48, 831–853 10.1016/j.neuropsychologia.2010.01.001 20074580

[B44] Greve A. Evans C. J. Graham K. S. Wilding E. L. (2011). Functional specialisation in the hippocampus and perirhinal cortex during the encoding of verbal associations. Neuropsychologia 49, 2746–2754 10.1016/j.neuropsychologia.2011.06.002 21683723

[B45] Griffiths T. D. (2001). The neural processing of complex sounds. Ann. N. Y. Acad. Sci. 930, 133–142 10.1111/j.1749-6632.2001.tb05729.x 11458824

[B46] Grill-Spector K. Henson R. Martin A. (2006). Repetition and the brain: neural models of stimulus-specific effects. Trends Cogn. Sci. (Regul. Ed.) 10, 14–23 10.1016/j.tics.2005.11.006 16321563

[B47] Hébert S. Peretz I. (2001). Are text and tune of familiar songs separable by brain damage? Brain Cogn. 46, 169–175 10.1016/S0278-2626(01)80058-0 11527321

[B48] Hein G. Knight R. T. (2008). Superior temporal sulcus-it’s my area: or is it? J. Cogn. Neurosci. 20, 2125–2136 10.1162/jocn.2008.20148 18457502

[B49] Hickok G. Houde J. Rong F. (2011). Sensorimotor integration in speech processing: computational basis and neural organization. Neuron 69, 407–422 10.1016/j.neuron.2011.01.019 21315253 PMC3057382

[B50] Hickok G. Poeppel D. (2007). The cortical organization of speech processing. Nat. Rev. Neurosci. 8, 393–402 10.1038/nrn2113 17431404

[B51] Johnson S. K. Halpern A. R. (2012). Semantic priming of familiar songs. Mem. Cognit. 40, 579–593 10.3758/s13421-011-0175-z 22227862

[B52] Kiebel S. J. Daunizeau J. Friston K. J. (2008). A hierarchy of time-scales and the brain. PLoS Comput. Biol. 4:e1000209 10.1371/journal.pcbi.1000209 19008936 PMC2568860

[B53] Koelsch S. (2012). Brain and Music. West Sussex: Wiley-Blackwell

[B54] Krekelberg B. Boynton G. Vanwezel R. (2006). Adaptation: from single cells to bold signals. Trends Neurosci. 29, 250–256 10.1016/j.tins.2006.02.008 16529826

[B55] Lebrun-Guillaud G. Tillmann B. Justus T. (2008). Perception of tonal and temporal structures in chord sequences by patients with cerebellar damage. Music Percept. 25, 271–283 10.1525/mp.2008.25.4.271

[B56] Lee A. C. (2006). Differentiating the roles of the hippocampus and perirhinal cortex in processes beyond long-term declarative memory: a double dissociation in dementia. J. Neurosci. 26, 5198–5203 10.1523/JNEUROSCI.3157-05.2006 16687511 PMC6674247

[B57] Lee A. C. Bussey T. J. Murray E. A. Saksida L. M. Epstein R. A. Kapur N. (2005). Perceptual deficits in amnesia: challenging the medial temporal lobe ‘mnemonic’ view. Neuropsychologia 43, 1–11 10.1016/j.neuropsychologia.2004.07.017 15488899

[B58] Lee A. C. Rudebeck S. R. (2010a). Human medial temporal lobe damage can disrupt the perception of single objects. J. Neurosci. 30, 6588–6594 10.1523/JNEUROSCI.0116-10.2010 20463221 PMC3079896

[B59] Lee A. C. Rudebeck S. R. (2010b). Investigating the interaction between spatial perception and working memory in the human medial temporal lobe. J. Cogn. Neurosci. 22, 2823–2835 10.1162/jocn.2009.21396 19925184 PMC2929461

[B60] Lehéricy S. Cohen L. Bazin B. Samson S. Giacomini E. Rougetet R. (2000). Functional MR evaluation of temporal and frontal language dominance compared to the Wada test. Neurology 54, 1625–1633 10.1212/WNL.54.8.1625 10762504

[B61] Liao W. Zhang Z. Pan Z. Mantini D. Ding J. Duan X. (2011). Default mode network abnormalities in mesial temporal lobe epilepsy: a study combining fMRI and DTI. Hum. Brain Mapp. 32, 883–895 10.1002/hbm.21076 20533558 PMC6870458

[B62] Liebenthal E. (2005). Neural substrates of phonemic perception. Cereb. Cortex 15, 1621–1631 10.1093/cercor/bhi040 15703256

[B63] Loui P. Wu E. H. Wessel D. L. Knight R. T. (2009). A generalized mechanism for perception of pitch patterns. J. Neurosci. 29, 454–459 10.1523/JNEUROSCI.4503-08.2009 19144845 PMC2779050

[B64] McClelland J. L. McNaughton B. L. O’Reilly R. C. (1995). Why there are complementary learning systems in the hippocampus and neocortex: insights from the successes and failures of connectionist models of learning and memory. Psychol. Rev. 102, 419 7624455 10.1037/0033-295X.102.3.419

[B65] Merrill J. Sammler D. Bangert M. Goldhahn D. Lohmann G. Turner R. (2012). Perception of words and pitch patterns in song and speech. Front. Psychol. 3:76 10.3389/fpsyg.2012.00076 22457659 PMC3307374

[B66] Meyer P. Mecklinger A. Grunwald T. Fell J. Elger C. E. Friederici A. D. (2005). Language processing within the human medial temporal lobe. Hippocampus 15, 451–459 10.1002/hipo.20070 15714509

[B67] Mishkin M. (1982). A memory system in the monkey. Philos. Trans. R. Soc. Lond. B Biol. Sci. 298, 85–95 10.1098/rstb.1982.0074 6125978

[B68] Möttönen R. Calvert G. A. Jääskeläinen I. P. Matthews P. M. Thesen T. Tuomainen J. (2006). Perceiving identical sounds as speech or non-speech modulates activity in the left posterior superior temporal sulcus. Neuroimage 30, 563–569 10.1016/j.neuroimage.2005.10.002 16275021

[B69] Noppeney U. Price C. J. (2004). An fMRI study of syntactic adaptation. J. Cogn. Neurosci. 16, 702–713 10.1162/089892904323057399 15165357

[B70] Oldfield R. C. (1971). The assessment and analysis of handedness: the Edinburgh inventory. Neuropsychologia 9, 97–113 5146491 10.1016/0028-3932(71)90067-4

[B71] Olsen R. K. Moses S. N. Riggs L. Ryan J. D. (2012). The hippocampus supports multiple cognitive processes through relational binding and comparison. Front. Hum. Neurosci. 6:146 10.3389/fnhum.2012.00146 22661938 PMC3363343

[B72] Opitz B. (2010). Neural binding mechanisms in learning and memory. Neurosci. Biobehav. Rev. 34, 1036–1046 10.1016/j.neubiorev.2009.11.001 19914286

[B73] O’Reilly R. C. Rudy J. W. (2001). Conjunctive representations in learning and memory: principles of cortical and hippocampal function. Psychol. Rev. 108, 311–345 10.1037/0033-295X.108.2.311 11381832

[B74] Overath T. Cusack R. Kumar S. Kriegstein K. von Warren J. D. Grube M. (2007). An information theoretic characterisation of auditory encoding. PLoS Biol. 5:e288 10.1371/journal.pbio.0050288 17958472 PMC2039771

[B75] Overath T. Kumar S. Kriegstein K. von Griffiths T. D. (2008). Encoding of spectral correlation over time in auditory cortex. J. Neurosci. 28, 13268–13273 10.1523/JNEUROSCI.4596-08.2008 19052218 PMC3844743

[B76] Parsons L. M. (2001). Exploring the functional neuroanatomy of music performance, perception, and comprehension. Ann. N. Y. Acad. Sci. 930, 211–231 10.1111/j.1749-6632.2001.tb05735.x 11458831

[B77] Patel A. (2008). Music, Language, and the Brain. New York: Oxford University Press

[B78] Patterson R. D. Uppenkamp S. Johnsrude I. S. Griffiths T. D. (2002). The processing of temporal pitch and melody information in auditory cortex. Neuron 36, 767–776 10.1016/S0896-6273(02)01060-7 12441063

[B79] Peretz I. Coltheart M. (2003). Modularity of music processing. Nat. Neurosci. 6, 688–691 10.1038/nn1083 12830160

[B80] Peretz I. Gagnon L. Hebert S. Macoir J. (2004). Singing in the brain: insights from cognitive neuropsychology. Music Percept. 21, 373–390 10.1525/mp.2004.21.3.373

[B81] Peynircioglu Z. F. Rabinovitz B. E. Thompson J. L. (2008). Memory and metamemory for songs: the relative effectiveness of titles, lyrics, and melodies as Cues for each other. Psychol. Music 36, 47–61 10.1177/0305735607079722

[B82] Reuter M. Schmansky N. J. Rosas H. D. Fischl B. (2012). Within-subject template estimation for unbiased longitudinal image analysis. Neuroimage 61, 1402–1418 10.1016/j.neuroimage.2012.02.084 22430496 PMC3389460

[B83] Rey A. (1964). L’Examen Clinique en Psychologie. Paris: Presses Universitaires de France

[B84] Riley J. D. Franklin D. L. Choi V. Kim R. C. Binder D. K. Cramer S. C. (2010). Altered white matter integrity in temporal lobe epilepsy: association with cognitive and clinical profiles. Epilepsia 51, 536–545 10.1111/j.1528-1167.2009.02508.x 20132296 PMC2929974

[B85] Robine M. (1994). Anthologie de la chanson française—des trouvères aux grands auteurs du XIXe siècle. Paris: Albin Michel

[B86] Rolls E. T. (1996). A theory of hippocampal function in memory. Hippocampus 6, 601–620 9034849 10.1002/(SICI)1098-1063(1996)6:6<601::AID-HIPO5>3.0.CO;2-J

[B87] Rose N. S. Olsen R. K. Craik F. I. Rosenbaum R. S. (2012). Working memory and amnesia: the role of stimulus novelty. Neuropsychologia 50, 11–18 10.1016/j.neuropsychologia.2011.10.016 22044651

[B88] Rudy J. W. Sutherland R. J. (1995). Configural association theory and the hippocampal formation: an appraisal and reconfiguration. Hippocampus 5, 375–389 8773252 10.1002/hipo.450050502

[B89] Saito Y. Ishii K. Sakuma N. Kawasaki K. Oda K. Mizusawa H. (2012). Neural substrates for semantic memory of familiar songs: is there an interface between lyrics and melodies? PLoS ONE 7:e46354 10.1371/journal.pone.0046354 23029492 PMC3460812

[B90] Sammler D. Baird A. Valabregue R. Clement S. Dupont S. Belin P. (2010). The relationship of lyrics and tunes in the processing of unfamiliar songs: a functional magnetic resonance adaptation study. J. Neurosci. 30, 3572–3578 10.1523/JNEUROSCI.2751-09.2010 20219991 PMC6632242

[B91] Samson S. Zatorre R. J. (1991). Recognition memory for text and melody of songs after unilateral temporal lobe lesion: evidence for dual encoding. J. Exp. Psychol. Learn. Mem. Cogn. 17, 793–804 1832437 10.1037//0278-7393.17.4.793

[B92] Samson S. Zatorre R. J. (1992). Learning and retention of melodic and verbal information after unilateral temporal lobectomy. Neuropsychologia 30, 815–826 1407496 10.1016/0028-3932(92)90085-z

[B93] Saur D. Kreher B. W. Schnell S. Kummerer D. Kellmeyer P. Vry M.-S. (2008). Ventral and dorsal pathways for language. Proc. Natl. Acad. Sci. U.S.A. 105, 18035–18040 10.1073/pnas.0805234105 19004769 PMC2584675

[B94] Schön D. Gordon R. Campagne A. Magne C. Astésano C. Anton J. (2010). Similar cerebral networks in language, music and song perception. Neuroimage 51, 450–461 10.1016/j.neuroimage.2010.02.023 20156575

[B95] Schön D. Gordon R. L. Besson M. (2005). Musical and linguistic processing in song perception. Ann. N. Y. Acad. Sci. 1060, 71–81 10.1196/annals.1360.006 16597752

[B96] Scott S. K. Blank C. C. Rosen S. Wise R. J. (2000). Identification of a pathway for intelligible speech in the left temporal lobe. Brain 123, 2400–2406 10.1093/brain/123.12.2400 11099443 PMC5630088

[B97] Scott S. K. Johnsrude I. S. (2003). The neuroanatomical and functional organization of speech perception. Trends Neurosci. 26, 100–107 10.1016/S0166-2236(02)00037-1 12536133

[B98] Scoville W. B. Milner B. (1957). Loss of recent memory after bilateral hippocampal lesions. J. Neurol. Neurosurg. Psychiatr. 20, 11–21 13406589 10.1136/jnnp.20.1.11 PMC497229

[B99] Serafine M. Crowder R. G. Repp B. (1984). Integration of melody and text in memory for songs. Cognition 16, 285–303 6541107 10.1016/0010-0277(84)90031-3

[B100] Serafine M. Davidson J. Crowder R. G. Repp B. (1986). On the nature of melody-text integration in memory for songs. J. Mem. Lang. 25, 123–135

[B101] Shimamura A. P. (2010). Hierarchical relational binding in the medial temporal lobe: the strong get stronger. Hippocampus 20, 1206–1216 10.1002/hipo.20856 20824723

[B102] Staresina B. P. Davachi L. (2009). Mind the gap: binding experiences across space and time in the human hippocampus. Neuron 63, 267–276 10.1016/j.neuron.2009.06.024 19640484 PMC2726251

[B103] Stoodley C. Schmahmann J. (2009). Functional topography in the human cerebellum: a meta-analysis of neuroimaging studies. Neuroimage 44, 489–501 10.1016/j.neuroimage.2008.08.039 18835452

[B104] Suarez R. O. Golby A. Whalen S. Sato S. Theodore W. H. Kufta C. V. (2010). Contributions to singing ability by the posterior portion of the superior temporal gyrus of the non-language-dominant hemisphere: first evidence from subdural cortical stimulation, Wada testing, and fMRI. Cortex 46, 343–353 10.1016/j.cortex.2009.04.010 19570530 PMC2821975

[B105] Sutherland R. J. Rudy J. W. (1989). Configural association theory: the role of the hippocampal formation in learning, memory, and amnesia. Psychobiology 17, 129–144

[B106] Suzuki W. A. (2009). Perception and the medial temporal lobe: evaluating the current evidence. Neuron 61, 657–666 10.1016/j.neuron.2009.02.009 19285462

[B107] Suzuki W. A. Baxter M. G. (2009). Memory, perception, and the medial temporal lobe: a synthesis of opinions. Neuron 61, 678–679 10.1016/j.neuron.2009.02.009 19285464

[B108] Sziklas V. Jones-Gotman M. (2008). RAVLT and nonverbal analog: French forms and clinical findings. Can. J. Neurol. Sci. 35, 323–330 18714800 10.1017/s0317167100008908

[B109] Thivard L. Hombrouck J. Tézenas du Montcel S. Delmaire C. Cohen L. Samson S. (2005). Productive and perceptive language reorganisation in temporal lobe epilepsy. Neuroimage 24, 841–851 10.1016/j.neuroimage.2004.10.001 15652319

[B110] Tierney A. Dick F. Deutsch D. Sereno M. (2012). Speech versus song: multiple pitch-sensitive areas revealed by a naturally occurring musical illusion. Cereb. Cortex 23, 255–263 10.1093/cercor/bhs003 22314043 PMC3539450

[B111] Tillmann B. Justus T. Bigand E. (2008). Cerebellar patients demonstrate preserved implicit knowledge of association strengths in musical sequences. Brain Cogn. 66, 161–167 10.1016/j.bandc.2007.07.005 17881108 PMC2271057

[B112] Turk-Browne N. B. Yi D.-J. Chun M. M. (2006). Linking implicit and explicit memory: common encoding factors and shared representations. Neuron 49, 917–927 10.1016/j.neuron.2006.01.030 16543138

[B113] Wagner K. Frings L. Spreer J. Buller A. Everts R. Halsband U. (2008). Differential effect of side of temporal lobe epilepsy on lateralization of hippocampal, temporolateral, and inferior frontal activation patterns during a verbal episodic memory task. Epilepsy Behav. 12, 382–387 10.1016/j.yebeh.2007.11.003 18158273

[B114] Winters B. D. (2004). Double dissociation between the effects of peri-postrhinal cortex and hippocampal lesions on tests of object recognition and spatial memory: heterogeneity of function within the temporal lobe. J Neurosci. 24, 5901–5908 10.1523/JNEUROSCI.1346-04.2004 15229237 PMC6729235

[B115] Yasuda C. L. Valise C. Saúde A. V. Pereira A. R. Pereira F. R. Ferreira Costa A. L. (2010). Dynamic changes in white and gray matter volume are associated with outcome of surgical treatment in temporal lobe epilepsy. Neuroimage 49, 71–79 10.1016/j.neuroimage.2009.08.014 19683060

[B116] Zarahn E. (2004). Positive evidence against human hippocampal involvement in working memory maintenance of familiar stimuli. Cereb. Cortex 15, 303–316 10.1093/cercor/bhh132 15342440

[B117] Zola-Morgan S. Squire L. R. (1993). Neuroanatomy of memory. Annu. Rev. Neurosci. 16, 547–563 10.1146/annurev.ne.16.030193.002555 8460903

